# Osteoarticular Tuberculosis of the Knee as a Unique Presentation in a 10-month-old Infant: A Rare Case of a Commonly Delayed Diagnosis

**DOI:** 10.1055/s-0042-1748944

**Published:** 2022-06-02

**Authors:** Carlos Gottschalk, Emanuela da Rocha Carvalho

**Affiliations:** 1 Departamento de Pediatria da Universidade Federal de Santa Catarina, Florianópolis, SC, Brasil; 2 Departamento de Infectologia Pediátrica do Hospital Infantil Joana de Gusmão, Florianópolis, SC, Brasil

**Keywords:** arthritis, knee joint, tuberculosis, osteoarticular

## Abstract

Osteoarticular tuberculosis of the knee is an uncommon disease presentation, especially in children under 1 year old. Diagnosis based on classic methods (such as culture and anatomopathological examination) is a challenge due to the paucibacillary characteristic of the infection. Risk factors include contact with individuals with bacilliferous tuberculosis, living in a region with high disease prevalence, and pediatric age group. We describe a case of chronic monoarthritis caused by *Mycobacterium tuberculosis* and intermittent inflammatory manifestations in a 10-month-old male patient with no extra-articular symptoms and no history of contact with bacilliferous tuberculosis. The culture was negative, and the anatomopathological examination was inconclusive for the etiologic agent. The detection of traces of *M. tuberculosis* DNA by a rapid molecular test (GeneXpert) based on the polymerase chain reaction technique established the diagnosis. The treatment consisted of antituberculosis drugs and led to complete resolution of the clinical-radiographic picture. This case emphasizes the importance of considering tuberculosis in the initial differential etiologic diagnoses of arthritis and, therefore, the need for an early, specific investigation, even when the clinical suspicion is not high.

## Introduction

Tuberculosis is an infectious disease caused by the bacillus *Mycobacterium tuberculosis* . It is a major cause of death by a single organism and one of the 10 most important causes of death worldwide. 1 Brazil is deemed endemic and a priority for disease control because it is among the 30 countries with the highest tuberculosis burden around the globe. 1 Arthritis is a rare form of tuberculosis, with an incidence ranging from 1 to 2% of the general population and the pediatric age group. 2 Diagnosis is often delayed due to the non-specificity of clinical-radiographic aspects and complex identification of the etiologic agent, increasing the chances of complications. 2 3 Considering the rarity of the presentation and the importance of an early diagnosis, we present this observational, descriptive study as a case report of knee joint tuberculosis. The child's guardian consented with the study, which was approved by the ethics committee.

## Case report

A 10-month-old male child, accompanied by his parents, presented to the orthopedic emergency of a tertiary hospital on June 8, 2018, with a history of pain, movement limitation, and intermittent swelling in the right knee for 6 months. During this period, further investigation excluded septic arthritis, and there was suspicion of a rheumatologic cause. The vaccination schedule was up to date. Family members denied fever, chills, cough, weight loss, or respiratory or systemic symptoms.

The patient was referred to the rheumatology outpatient clinic. There was only mild discomfort in the right knee, sporadic quarterly episodes of edema and movement limitation, and no other complaints. The rheumatological markers were negative, with a slight increase in the erythrocyte sedimentation rate (ESR) and C-reactive protein (CRP) levels, as shown in Box 1 . On January 24, 2018, a magnetic resonance imaging (MRI) of the right knee revealed significant joint effusion, synovitis, lymph node enlargement in the posterior region, and subcutaneous edema on the anterior surface. Boxes 2 and 3 describe the radiological evolution.

**Box 1:** Evolution of inflammatory markers (erythrocyte sedimentation rate [ESR] and C-reactive protein [CRP])

Date	ESR	CRP
December 12, 2017	33	0
December 14, 2017	18	0
December 21, 2017	60	43.92
February 1, 2018	7	0.6
June 7, 2018	30	9.2
December 11, 2018	30	0.8
February 26, 2019	15	0.4
June 7, 2019	15	0.001
August 21, 2019	10	0.001
October 9, 2019	15	1.67
November 29, 2019	13	0.25
January 28, 2020	22	6.31
February 6, 2020	55	11
February 27, 2020	25	2.9

**Box 2:** Radiological evolution at right knee ultrasound

Date	Right knee ultrasound
December 09, 2016	Synovial thickening stretching the suprapatellar synovial recess. Enlarged lymph nodes in the popliteal fossa.
December 12, 2017	Heterogeneous joint effusion associated with synovial thickening. Enlarged lymph nodes in the popliteal fossa.
April 27, 2018	Moderate joint effusion associated with synovial thickening, consistent with synovitis. Lymph nodes in the popliteal fossa
July 27, 2019	Presence of small/moderate joint effusion associated with synovial thickening, with no defined flow on Doppler evaluation.

**Box 3:** Radiological evolution at right knee magnetic resonance imaging

Date	Right knee magnetic resonance imaging
January 24, 2018	Significant joint effusion with exuberant synovial thickening and contrast enhancement consistent with synovitis. Lymph nodes in the posterior region of the knee; the largest lymph node measures 1.1 × 1.7 cm. Edema in the subcutaneous tissue on the anterior aspect of the knee.
February 17, 2020	Large joint effusion with extensive synovitis and oval formations surrounded by inflammatory changes on the posterior aspect of the knee, in close contact with the joint capsule. In addition, there is adjacent lymph node enlargement and advanced popliteal tenosynovitis. These findings indicate septic arthritis, but they are not specific; consider reactivation of juvenile idiopathic arthritis as a differential diagnosis.
August 23, 2021	Complete resolution of joint effusion and synovitis compared to the previous examination. Resolution of lymph node enlargement in the popliteal fossa. There is a small, focal change in the subchondral bone signal in the load-bearing zone of the lateral femoral condyle, with a non-specific appearance; this finding must be followed-up per physician's discretion.

**Fig. 1:**
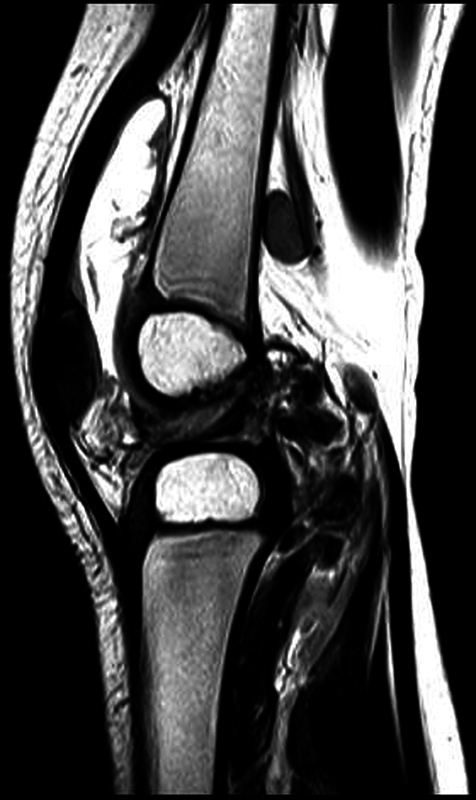
Sagittal, T1-weighted magnetic resonance image of the right knee (February 17, 2020) showing joint effusion, synovitis, enlarged lymph nodes, and oval formations in the posterior region.

**Fig. 2:**
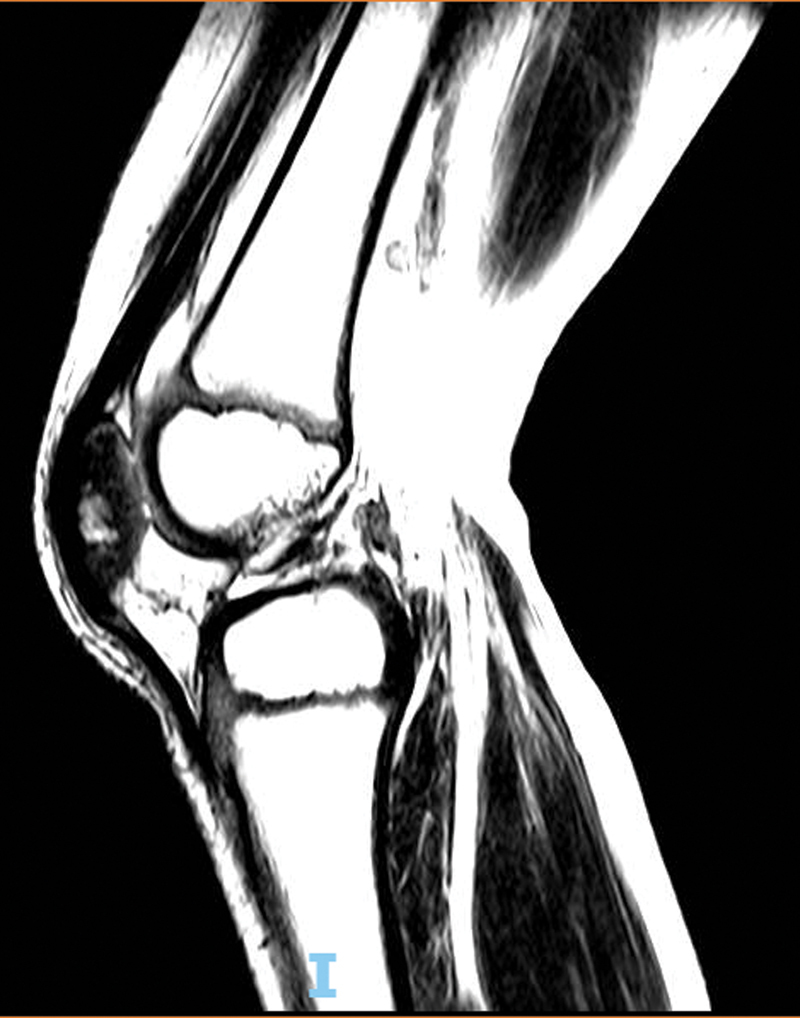
Sagittal, T1-weighted magnetic resonance image of the right knee (August 23, 2021) showing no abnormalities after tuberculosis treatment.

In this scenario, a probable diagnosis was juvenile idiopathic arthritis. The immunosuppressive treatment consisted of methotrexate, along with local steroid infiltrations during exacerbations. This clinical picture remained for 20 months despite increased drug dosage and administration of cyclosporine. Due to recent availability, the patient underwent a tuberculin skin test (with purified protein derivative, PPD) on February 3, 2020; the positive result (17 mm) led to the suspicion of tuberculosis. There was no identification of contact with bacilliferous tuberculosis. On February 17, 2020, an MRI showed persistent joint effusion, synovitis, and lymph node enlargement, now accompanied by oval formations ( Fig. 1 ). An open biopsy performed on March 10, 2020, revealed granulomatous tissue. Culture results were negative. The GeneXpert molecular test identified traces of *M. tuberculosis* DNA. Treatment consisted of dispersible tablets recommended by the Ministry of Health for children younger than 10 years old, with the administration of isoniazid and rifampicin for 12 months, plus pyrazinamide during the first 2 months. Symptomatic resolution occurred 2 months after the beginning of treatment, and an MRI on August 23, 2021, showed radiological improvement of the lesions ( Fig. 2 ).

## Discussion

This case demonstrates the importance of tuberculosis as a differential diagnosis of arthritis, the challenges of identifying the etiologic agent, and the need for its early investigation to avoid severe sequelae and deformities.

Involvement occurs by direct invasion of the joint space by *M. tuberculosis* , followed by lymphohematogenous dissemination resulting from a latent primary infection or caused by an inflammatory reaction in an extraarticular focus. 2 The clinical picture features episodes of pain, edema, and decreased joint range of motion with partial or complete resolution within weeks, and no systemic manifestations. The insidious course and intermittent symptoms make the clinical picture indistinguishable from other forms of subacute or chronic arthritis. 2 4

Laboratory findings include a slight increase in inflammatory markers, such as CRP and ESR. 2 The ultrasonography identified joint effusion and assisted in specimen collection. An MRI provides a more detailed analysis, potentially indicating bone marrow lesions, joint effusion, synovitis, bone or cartilaginous erosions, and joint space reduction. 5 Radiographic evolution consists of local osteopenia and potential soft-tissue edema, progressing to one or more areas of bone erosion; eventually, there is joint space reduction, with or without anatomical disorganization. This sequence relates to the duration of the disease and the patient's immune response. 6

An aspiration puncture or biopsy is often required because of the clinical limitation and the lack of supplementary tests. 2 3 7 Traditionally, observation of caseous granuloma in histological analysis or a positive culture confirms the diagnosis. *M. tuberculosis* identification using classical methods is difficult due to the paucibacillary feature of the extrapulmonary manifestations, challenges in lesion access, and the limited amount of specimen. Tests based on polymerase chain reaction techniques, such as the fast molecular test, have shown high diagnostic efficiency due to speed and good sensitivity regardless of the specimen. 7 8 The tuberculin skin test is positive in most immunocompetent patients with tuberculosis-related arthritis. Despite being a simple procedure, it is essential and should be part of the initial investigation of arthritis, especially in endemic regions. In our case, the late performance of this test was due to a short supply of PPD from 2014 to 2018. 9

Most patients respond well to early treatment. The treatment is surgical for severe cases with significant joint space reduction or major anatomical alterations. For children under 10 years old, the currently indicated regimen consists of isoniazid, rifampicin, and pyrazinamide administration for 2 months, followed by isoniazid and rifampicin for 10 months as maintenance treatment. Drugs are given as dispersible tablets to improve therapeutic adherence. 10 Local symptoms usually regress completely after 2 months of treatment, and a radiographic improvement is noticeable in about 6 weeks.

Knee arthritis is an uncommon presentation of extrapulmonary tuberculosis in infants. Diagnosis is difficult and often late due to the non-specific clinical picture and supplementary test results. Non-surgical treatment is effective in the early stages of the disease. To avoid serious complications of the disease, tuberculosis should be part of the early differential diagnosis of arthritis.
