# Supplementary material for: Osteoarticular Tuberculosis of the Knee as a Unique Presentation in a 10-month-old Infant: A Rare Case of a Commonly Delayed Diagnosis
Source: Rev Bras Ortop (Sao Paulo). 2022 Jun 2;59(Suppl 1):e56–9. doi: 10.1055/s-0042-1748944 (PMC11254435; doi:10.1055/s-0042-1748944)
Supplement: Related texts

# Research article: Tuberculose osteoarticular do joelho como apresentação única em lactente de 10 meses: Um caso raro de diagnóstico comumente tardio

**Keywords:** articulação do joelho, artrite, tuberculose osteoarticular

## Abstract

A tuberculose osteoarticular do joelho é uma apresentação incomum da doença, especialmente em crianças com menos de 1 ano de idade. A característica paucibacilar da infecção torna o diagnóstico um desafio, levando em consideração métodos mais clássicos como a cultura e o anatomopatológico. Os fatores de risco são contato com indivíduos com tuberculose bacilífera, estar em uma região de alta prevalência, e populações pediátricas. O presente relato descreve um caso de monoartrite por *Mycobacterium tuberculosis* , de curso crônico e manifestações inflamatórias intermitentes em um paciente masculino de 10 meses, sem sintomatologia extra-articular e sem história de convívio ou contato prévio com tuberculose bacilífera. A cultura foi negativa e o exame anatomopatológico foi inconclusivo para o agente etiológico e o diagnóstico foi realizado pela detecção de traços de DNA de *M. tuberculosis* no teste rápido molecular (GeneXpert), utilizando a técnica da reação em cadeia da polimerase. O tratamento foi realizado com medicamentos antituberculose e houve resolução completa do quadro clínico-radiográfico. Este caso enfatiza a importância de considerar a tuberculose como parte dos diagnósticos etiológicos diferenciais iniciais das artrites e, portanto, a necessidade da investigação precoce específica a esta bactéria, ainda que a suspeição clínica não seja elevada.

## Introdução

A tuberculose é a doença infectocontagiosa causada pelo bacilo *M. tuberculosis* . É a maior causa de morte por um agente infeccioso único e uma das 10 principais causas de morte no mundo. 1 O Brasil é apontado como endêmico e está entre os 30 países de alta carga para tuberculose no mundo, sendo prioritário para o controle da doença. 1 A artrite é uma forma rara com incidência de 1 a 2% na população geral e na faixa pediátrica. 2 Os aspectos clínicos-radiográficos são inespecíficos e a identificação do agente etiológico complexa, tornando comum o diagnóstico tardio e aumentando as chances de complicação. 2 3 Considerando a raridade da apresentação e a importância do diagnóstico precoce, apresentamos este estudo observacional e descritivo do tipo relato de caso de tuberculose articular do joelho. Houve consentimento dos responsáveis e aprovação em comitê de ética.

## Relato de caso

Um paciente do sexo masculino, com 10 meses de idade, acompanhado pelos pais, apresentou-se à emergência ortopédica de um hospital terciário em 08/06/2018 com quadro de dor, limitação de movimento e edema intermitente em joelho direito há 6 meses. Nesse período, após investigação complementar, foi excluída artrite séptica e suspeitada causa reumatológica. O calendário vacinal estava atualizado. Os familiares negaram história prévia de febre, calafrios, tosse, perda de peso ou quaisquer sintomas respiratórios ou sistêmicos.

O paciente foi então encaminhado ao ambulatório de reumatologia. Mantinha-se apenas com leve desconforto em joelho direito, episódios esporádicos trimestrais de edema e limitação de movimento, sem outras queixas. Os marcadores reumatológicos eram negativos, apenas discreto aumento da velocidade de hemossedimentação (VHS) e da proteína C-reativa (PCR), conforme o Quadro 1 . A ressonância magnética (RNM) do joelho direito de 24/01/2018 descreve importante derrame articular, sinovite, linfonodomegalias em região posterior e edema de subcutâneo na face anterior. Os Quadros 2 e 3 descrevem a evolução radiográfica.

**Quadro 1:** Evolução das provas inflamatórias (VHS e PCR)

Data	VHS	PCR
12/12/2017	33	0
14/12/2017	18	0
21/21/2017	60	43,92
01/02/2018	7	0,6
07/06/2018	30	9,2
11/12/2018	30	0,8
26/02/2019	15	0,4
07/06/2019	15	0,001
21/08/2019	10	0,001
09/10/2019	15	1,67
29/11/2019	13	0,25
28/01/2020	22	6,31
06/02/2020	55	11
27/02/2020	25	2,9

Abreviaturas: PCR, proteína C-reativa; VHS, velocidade de hemossedimentação.

**Quadro 2:** Evolução radiográfica pela ultrassonografia do joelho direito

Data	Ultrassonografia do joelho direito
09/12/2016	Moderado espessamento sinovial distendendo o recesso sinovial suprapatelar. Linfonodomegalia na fossa poplítea.
12/12/2017	Derrame articular heterogêneo associado a espessamento sinovial. Linfonodomegalias na fossa poplítea.
27/04/2018	Moderado derrame articular associado a espessamento sinovial, compatível com sinovite. Linfonodos na fossa poplítea.
27/07/2019	Presença de derrame articular de pequenas/moderadas dimensões, associada a espessamento sinovial, sem fluxo definido ao doppler.

**Quadro 3:** Evolução radiográfica pela ressonância magnética do joelho direito

Data	Ressonância magnética do joelho direito
24/01/2018	Importante derrame articular, associado a exuberante espessamento sinovial com captação de contraste, compatível com sinovite. Linfonodos na região posterior do joelho, maior medindo 1,1 × 1,7 cm. Edema no tecido subcutâneo na face anterior do joelho.
17/02/2020	Volumoso derrame articular com extensa sinovite, associados a formações ovaladas envoltas por alterações inflamatórias localizadas no aspecto posterior do joelho, em íntimo contato com a cápsula articular, além de linfonodomegalias adjacentes e avançada tenossinovite do poplíteo. Estes achados formam a favor de artrite séptica, porém não são específicos, devendo ser considerada reativação de artrite idiopática juvenil no diagnóstico diferencial.
23/08/2021	Resolução completa do derrame articular e da sinovite em relação ao exame anterior. Resolução da linfonodomegalia na fossa poplítea. Na zona de apoio de carga do côndilo femoral lateral observa-se pequena alteração de sinal óssea subcondral, focal, de aspecto inespecífico, e a critério clínico manter sob controle.

Diante desse contexto, artrite idiopática juvenil foi considerada provável. Iniciou-se o tratamento imunossupressor com metotrexato, e foram realizadas infiltrações locais com corticoide em períodos de exacerbação. Por 20 meses, a clínica manteve-se apesar do aumento da dosagem e associação com ciclosporina. Pela recente disponibilidade, foi realizado um teste cutâneo tuberculínico (PPD) dia 03/02/2020, com resultado positivo (17 mm), iniciando a suspeita de tuberculose. Nenhum contato com tuberculose bacilífera foi identificado. Na RNM de 17/02/2020, o paciente mantinha-se com derrame articular, sinovite e linfonodomegalias, mas agora com formações ovaladas, vide Fig. 1 . A biópsia aberta realizada em 10/03/2020 revelou tecido granulomatoso e a cultura foi negativa; já o teste molecular GeneXpert identificou traços de DNA de *M. tuberculosis* . Iniciou-se esquema terapêutico com comprimidos dispersíveis preconizado pelo Ministério da Saúde para crianças menores de 10 anos, completando 12 meses de isoniazida e rifampicina, nos primeiros 2 meses acrescidos de pirazinamida. Após 2 meses do início do tratamento, houve resolução sintomática, e a RNM de 23/08/2021 demonstrou melhora radiológica das lesões, como mostra a Fig. 2 .

**Fig. 1:** Imagem de corte sagital da ressonância magnética do joelho direito (17/02/2020) em T1 mostrando derrame articular, sinovite, linfonodos aumentados e formações ovaladas em região posterior.

**Fig. 2:** Imagem de corte sagital da ressonância magnética do joelho direito (23/08/2021) em T1 sem alterações, após esquema terapêutico para tuberculose.

## Discussão

Este caso demonstra a importância de considerar a tuberculose como diagnóstico diferencial das artrites, os desafios da identificação do agente etiológico, e a necessidade de sua investigação precoce para evitar sequelas e deformidades graves.

O acometimento ocorre por invasão direta de *M. tuberculosis* no espaço articular associado a uma disseminação linfo-hematogênica decorrente de uma infecção primária latente ou causado por reação inflamatória a algum foco extra-articular. 2 A clínica envolve episódios de dor, edema, diminuição da amplitude de movimento articular que resolvem parcial ou completamente em semanas, sem manifestações sistêmicas. O curso insidioso com sintomatologia intermitente torna a clínica indistinguível de outras formas de artrites subagudas ou crônicas. 2 4

Os achados laboratoriais resumem-se em discreto aumento de provas inflamatórias, como a PCR e a VHS. 2 A ultrassonografia é utilizada para identificar derrame articular e auxiliar procedimentos de coleta de material. Para análises mais detalhadas, a RNM é o exame de escolha, podendo apontar lesões na medula óssea, derrame articular, sinovite, erosões ósseas ou cartilaginosas e diminuição do espaço articular. 5 A progressão radiográfica sucede com osteopenia local, possível edema de partes moles, evoluindo para uma ou mais áreas de erosão óssea e, por fim, diminuição do espaço articular, com ou sem desorganização anatômica. Esta sequência relaciona-se não só ao tempo de decorrência da doença, como também à resposta imunológica do paciente. 6

Tendo em vista a limitação da clínica e dos exames complementares, quase sempre é necessária uma punção aspirativa ou uma biópsia. 2 3 7 Tradicionalmente, a confirmação diagnóstica dá-se pela presença de granuloma caseoso em análise histológica ou pela cultura positiva. A característica paucibacilar das manifestações extrapulmonares, acesso difícil à lesão e a quantidade limitada de material dificultam a identificação do bacilo pelos métodos clássicos. Testes que utilizam a técnica da reação em cadeia da polimerase, como o teste rápido molecular têm demonstrado grande eficiência diagnóstica, tanto pela rapidez quanto pela boa sensibilidade, independente do material coletado. 7 8 A prova tuberculínica é positiva na maioria dos imunocompetentes com artrite tuberculosa. Apesar de ser um exame simples, é essencial e deve constar no início da investigação das artrites, principalmente em regiões endêmicas. No caso apresentado, a realização tardia deste exame decorreu do desabastecimento do PPD utilizado para realização do teste no período de 2014 a 2018. 9

A maioria dos pacientes tem boa resposta à terapia medicamentosa quando precoce, sendo as intervenções cirúrgicas reservadas aos quadros mais graves em que há diminuição expressiva do espaço articular ou alteração anatômica importante. Em menores de 10 anos, o regime indicado atualmente é a terapia com 2 meses com isoniazida, rifampicina e pirazinamida, seguidos da fase de manutenção de 10 meses com isoniazida e rifampicina na formulação de comprimidos dispersíveis, melhorando a adesão ao tratamento. 10 A sintomatologia local tende a regredir completamente com 2 meses de tratamento e a melhora radiográfica já é notável em cerca de 6 semanas.

A artrite do joelho é uma apresentação incomum da tuberculose extrapulmonar em lactentes, que pelo quadro clínico e exames complementares inespecíficos, torna o diagnóstico difícil e tardio na maioria dos casos. O tratamento não cirúrgico é eficaz nos estágios iniciais da doença. Para evitar as complicações graves da doença, a tuberculose deve ser considerada como diagnóstico diferencial de artrites a esclarecer e sua investigação deve ser realizada precocemente.
